# The mini chamber test: a novel bioassay for bioefficacy testing of chlorfenapyr treated nets used in malaria control

**DOI:** 10.12688/gatesopenres.16381.1

**Published:** 2026-04-08

**Authors:** Amy Lewis, Rowan Jones-Brown, Gemma Harvey, Giorgio Praulins, Emma Reid, Aidi Galus Lugenge, Frank Mechan, Katherine Gleave, Rosemary Susan Lees

**Affiliations:** 1 Liverpool School of Tropical Medicine, Liverpool, England, L3 5QA, UK; 2 Liverpool School of Tropical Medicine, Liverpool, England, L3 5QA, UK; 3 Ifakara Health Institute, Bagamoyo, P.O Box 74, Tanzania; 4 Liverpool School of Tropical Medicine, Liverpool, England, L3 5QA, UK

**Keywords:** Bioassays, chlorfenapyr, insecticide treated nets, bioefficacy, mosquito, Anopheles, vector control

## Abstract

Dual-active-ingredient insecticide-treated nets (Dual-AI ITNs) combining pyrethroids with chlorfenapyr are increasingly being deployed across malaria-endemic areas. Laboratory evaluation of chlorfenapyr ITNs is challenging due to chlorfenapyr’s requirement for metabolic activation.

Inspired by the tunnel test, the mini chamber test is smaller, has higher throughput, and is animal-free. We optimised the primary parameters using pyrethroid-susceptible and -resistant mosquito strains exposed to new pyrethroid-chlorfenapyr ITNs versus controls. With parameters defined, we used a draft standard operating procedure (SOP) to evaluate the mini chamber against the gold standard tunnel test in a comparator study. The generated data were used to finalise an SOP.

The number of mosquitoes per bioassay had no effect on 72 hour mortality on exposure to the pyrethroid-chlorfenapyr net (10, 20, or 30 mosquitoes per mini chamber: 98% vs. 99% vs. 99%, p = 0.46), nor did the chamber orientation (horizontal vs. vertical: 70% vs. 74%, p = 0.35). Mean 72-hour mortality reduced with the length of exposure: 99% with overnight exposure, 74% with 4 hours and 35% with 2 hour exposure. In direct comparisons, the mini chamber gave 95% 72 hour mortality and the tunnel test 70% with overnight exposure. A trend was observed between chemical content (measured by high performance liquid chromatography (HPLC)) and mortality in ITN samples that had been washed 20 times or used in experimental hut trials, compared to unused comparators, when a 6 hour exposure length was used, showing potential for application to durability monitoring.

A mini chamber SOP is shared ( https://github.com/i2i-Data-Repository/Mini-Chamber-Test-paper-data-R-code-and-SOP), and further improvements may come from internal and multi-site validation and review of data generated. The method shows promise as an effective bioassay for use where tunnel tests are not available, for measuring the biological activity of chlorfenapyr in ITNs when measuring regeneration times, wash resistance, residual efficacy, and other studies.

## Introduction

Despite the wide-scale rollout of vector control methods and the increase in availability of treatments, malaria remains a leading cause of morbidity and mortality worldwide, with an estimated 263 million cases and 597,000 deaths in 2023. ^1^ The large-scale deployment of vector control methods, including insecticide-treated nets (ITNs) and indoor residual spraying (IRS) across sub-Saharan Africa, has exerted substantial selection pressure, driving widespread insecticide resistance in mosquito vectors. Resistance increasingly threatens the efficacy of malaria control efforts owing to its rapid increase in intensity and geographic distribution, which reduces the killing capacity of ITNs and IRS. ^2^ The introduction of ITNs with a second active ingredient and a novel mode of action (dual-AI nets) is key to overcoming the increasing resistance.

These new insecticides have distinct modes of action to pyrethroids. Piperonyl-butoxide (PBO) acts synergistically to increase pyrethroid effectiveness by blocking cytochrome P450s and preventing detoxification. ^3^ Pyriproxyfen (PPF) is an insect growth regulator that inhibits egg production, thereby reducing fertility and fecundity. ^4^ Chlorfenapyr, a pyrrole pro-insecticide, is metabolised by cytochrome P450s to tralopyril, which interrupts oxidative phosphorylation in mitochondria and causes cell death and mosquito mortality. ^5^ Following successful epidemiological trials ^6^^,^^7^ the World Health Organisation (WHO) issued a strong recommendation for the deployment of pyrethroid-chlorfenapyr ITNs in areas with pyrethroid-resistant mosquitoes in December 2023. ^8^

Despite improved chemistry, ITNs face challenges such as net attrition, lack of community uptake, poor chemical durability, and behavioural resistance that limit their long-term field performance. ^9^ To ensure that ITNs maintain efficacy throughout their lifespan in the field, there is a pre-qualification process ^10^ review the safety, quality, and efficacy of new products. This includes reviewing data generated using bioassays, for example, to measure the chemical content and bioefficacy of ITN samples under conditions of artificial ageing. Current laboratory bioassays, such as the WHO cone test, are ill-suited for evaluating pro-insecticides, such as chlorfenapyr, that require metabolic activation. ^11^ Unlike the rapid neurotoxic effects of pyrethroids, chlorfenapyr requires metabolic activation, resulting in slower mosquito mortality and longer holding periods to reveal their full efficacy. Consequently, bioassays using chlorfenapyr ITNs require a 72-hour holding period compared to the standard 24-hour holding period post-exposure to pyrethroids. ^12^

In nature, mosquitoes are metabolically active when they encounter chlorfenapyr ITNs. Therefore, bioassays should replicate this to determine the full effects of chlorfenapyr. ^13^ The WHO currently recommends tunnel tests for ITN testing, except where rapid knockdown can be measured with a cone test ^14^ but these require small animals, posing logistical and ethical challenges in many laboratories. ^15^ This gap is addressed by the development of the mini chamber test. This method was designed to capture the delayed mortality effects of metabolically activated insecticides without the need for animal hosts.

Standardised bioassay protocols that are both scalable and produce reproducible data are essential for robust cross-country comparisons, regulatory approval, and post-market surveillance; however, such tools are currently lacking for the evaluation of ITNs treated with pro-insecticides. This study presents the optimisation of a novel laboratory bioassay, the mini chamber test, which was inspired by tunnel tests but without the requirement for an animal host and with increased throughput and ease of operation. Guided by the framework of Matope et al. (2023 ^16^), the method is characterised, test parameters are established, and a standard operating procedure (SOP) is drafted. The availability of the mini chamber will facilitate more robust data generation on chlorfenapyr-based ITN, for example, in the routine monitoring of residual efficacy and susceptibility of target populations, ultimately supporting better evidence-based deployment decisions in malaria control programmes.

## Methods

### Testing facility

The experimental work was conducted at two sites: The Liverpool Insect Testing Establishment (LITE) at the Liverpool School of Tropical Medicine (LSTM) and the Vector Control Product Testing Unit (VCPTU) of the Ifakara Health Institute (IHI) in Bagamoyo, Tanzania (6°26′ S, 38°53′ E).

### Mosquito rearing

Mosquito colonies were maintained in LITE at LSTM, following the methods described by Williams et al. (2019 ^17^). Insectary conditions were maintained at 27 ± 2°C and 70 ± 10% relative humidity (RH). Except where indicated, the mosquitoes were reared on a standard 12:12 light:dark cycle. As explained below, some tests were performed with ‘light shifted’ mosquitoes, reared with a reversed light cycle to allow for testing during the ‘night phase’ of the circadian rhythm which occurred from midday to midnight. The eggs were hatched in 500 ml of purified water (Millipore, Watford, UK). First-instar larvae were split on the day following hatching into large trays of ~600 larvae in 2.5 l of purified water. Larvae were fed ground TetraMin ^®^ tropical flakes (Tetra U.S., Blacksburg, VA, USA), six days a week. The larvae pupated approximately 8-10 days after the eggs hatched. Pupae were removed from the larval trays and placed in BugDorm rearing cages (MegaView Science, Taiwan) for eclosion. They were provided with continuous access to a 10% sucrose solution, except for the one-hour period before testing. For egg production, female adults were fed human blood using the Hemotek Membrane feeding system (Hemotek Ltd., Blackburn, UK). One day after the blood meal, an oviposition pot was added to the cages to collect the eggs.

Mosquito colonies at IHI, Tanzania, were maintained according to MR4 guidelines (MR4, 2023 ^18^). Mosquitoes were reared in an insectary at 27 ± 2°C and 40% - 100% relative humidity under an ambient 12:12 light: dark cycle. Larvae were maintained at an average density of 200 per litre and fed ground TetraMin ^®^ fish flakes (Tetra U.S., Blacksburg, VA, USA). Female adults were provided with a 10% sterile (autoclaved) sucrose solution and fed cow blood through a membrane feeding system to facilitate egg production.

### Mosquito strains

All mosquitoes used for testing were three to five-day old, non-blood fed females.

Several strains of pyrethroid-susceptible and pyrethroid-resistant *Anopheles* spp. have been used during method development. Resistance phenotypes were maintained through selection with deltamethrin every three-five generation using WHO susceptibility bioassays in LITE facilities. ^17^

An *An. arabiensis* pyrethroid resistant strain and an *An. gambiae* pyrethroid-susceptible strain were used for a comparison of the mini chambers and the WHO tunnel test at Ifakara Health Institute, Tanzania. Information regarding the strains used is presented in Table 1.

**Table 1.** Description of the mosquito strains used.

Species	Strain name	Pyrethroid resistance status	Mechanism of resistance	Origin	Years and place of colony establishment	Testing site used
*Anopheles gambiae*	Kisumu	Susceptible	NA	Kisumu, Kenya	1975, LSTM	LSTM
*Anopheles gambiae*	Tiassalé 13	Resistant	Upregulation of cytochrome P450s	Tiassalé, Côte d’Ivoire	2013, LSTM	LSTM
*Anopheles coluzzi*	Tiefora	Resistant	Upregulation of cytochrome P450s	Comoé Province, Burkina Faso	2018, LSTM	LSTM
*Anopheles arabiensis*	Kingani	Resistant	Upregulation of cytochrome P450s	Kingani, Tanzania	2006, IHI	IHI
*Anopheles gambiae*	Ifakara	Susceptible	NA	Ifakara, Tanzania	1996, IHI	IHI

LSTM = Liverpool School of Tropical Medicine, IHI = Ifakara Health Institute.

### Strain characterisation

In line with developing best practices, during the study, we started to characterise strains at the time of testing to confirm the resistance status of the strains used and to support the interpretation of the study results, according to the method of Lees et al. (2022 ^19^). After exposure to treated filter papers in a WHO tube test or ITN samples in a cone test, mosquitoes were aspirated into holding cups and mortality was measured 24 hours after exposure to a pyrethroid or 72 hours after exposure to chlorfenapyr. The results of this characterisation are listed in Table 5.

### ITN types

Four net types were used during the optimisation of the mini chamber test: an untreated net for the negative control, PermaNet 2.0, and the roof of PermaNet 3.0, for the pyrethroid-only and pyrethroid-PBO nets, respectively (Vestergaard Sarl, Switzerland), and PermaNet Dual as the pyrethroid-chlorfenapyr (Vestergaard Sarl, Switzerland). Whole nets were provided by Vestergaard Sarl and were stored at room temperature. The net types, active ingredients, and manufacturers are listed in Table 2.

**Table 2.** Description of the different net types used.

Net type	Net name	Active ingredient	Manufacturer
Untreated netting	N/A	N/A	N/A
Pyrethroid-only	PermaNet 2.0	Deltamethrin (1.4 g/kg ± 25%)	Vestergaard Sarl, Switzerland
Pyrethroid-PBO	PermaNet 3.0 roof	Deltamethrin (4 g/kg ± 25%) Piperonyl-butoxide (25 g/kg ± 25%)	Vestergaard Sarl, Switzerland
Pyrethroid-chlorfenapyr	PermaNet Dual	Deltamethrin (2.1 g/kg ± 25%) Chlorfenapyr (5 g/kg ± 25%)	Vestergaard Sarl, Switzerland

**Figure 1.**
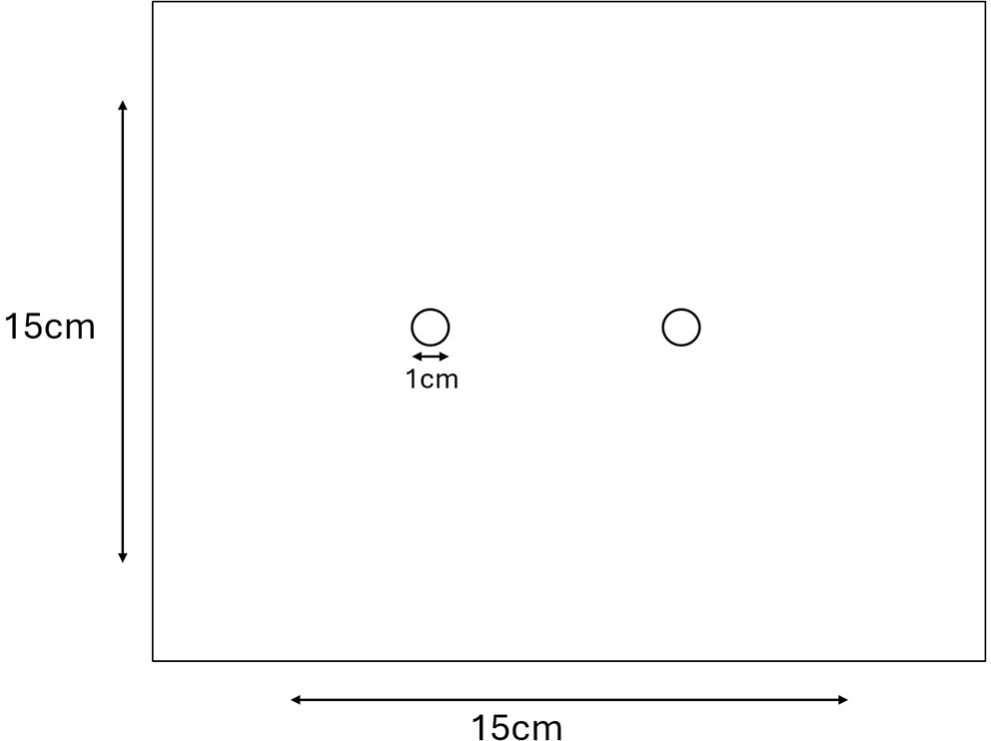
Size of the net pieces and holes in the net pieces used in the mini chamber test.

To evaluate the method, all pieces of each net type were cut from the same full net to limit batch variability. A total of 55 samples measuring 18 × 18 cm were cut from the roof section, and an additional 58 PermaNet Dual samples measuring 15 × 15 cm were cut from a side panel once the roof panel was used. The change in the size of the pieces allowed easier construction of the chambers. Net pieces were aired at 27°C for at least 7 days, then individually wrapped in tin foil and stored at 4°C until testing. Two holes, one cm in diameter were cut in the middle of each square ( Figure 1). The net pieces were wrapped in foil and stored at 4°C until high performance liquid chromatography (HPLC) was performed.

In addition, samples of the pyrethroid-chlorfenapyr net, Interceptor ^®^ G2, were obtained from Ifakara Health Institute (IHI). These were either unwashed and unused, unwashed and used in an experimental hut study, or washed and used in an experimental hut study ( Table 3). The net samples were wrapped in foil for storage after testing and shipping to LSTM.

**Table 3.** Description of secondary net types used.

Net name	Active ingredient	Washes	Previous uses
Baseline	Alpha-cypermethrin (100 mg/m ^2^ ± 25%) Chlorfenapyr (200 mg/m ^2^ ± 25%)	0	0
Hut used	Alpha-cypermethrin (100 mg/m ^2^ ± 25%) Chlorfenapyr (200 mg/m ^2^ ± 25%)	0	6-8 months in an experimental hut trial
Hut washed	Alpha-cypermethrin (100 mg/m ^2^ ± 25%) Chlorfenapyr (200 mg/m ^2^ ± 25%)	20	6-8 months in an experimental hut trial

### Mini chamber design

Two versions of the mini chamber were tested in this study. The initial version of the mini chamber was adapted from a commercial rearing cage, Insect Breeder Pots, measuring 10.5 × 9.5 × 7.5 cm, made by BugDorm (MegaView Science, Taiwan). The funnel between the two compartments was removed using a soldering iron, leaving the connectors to hold the compartments together. The net pieces were held between the two chambers using a connector ring. Parafilm was wrapped around the outside during testing to secure the equipment in place and to prevent escapees.

**Figure 2.**
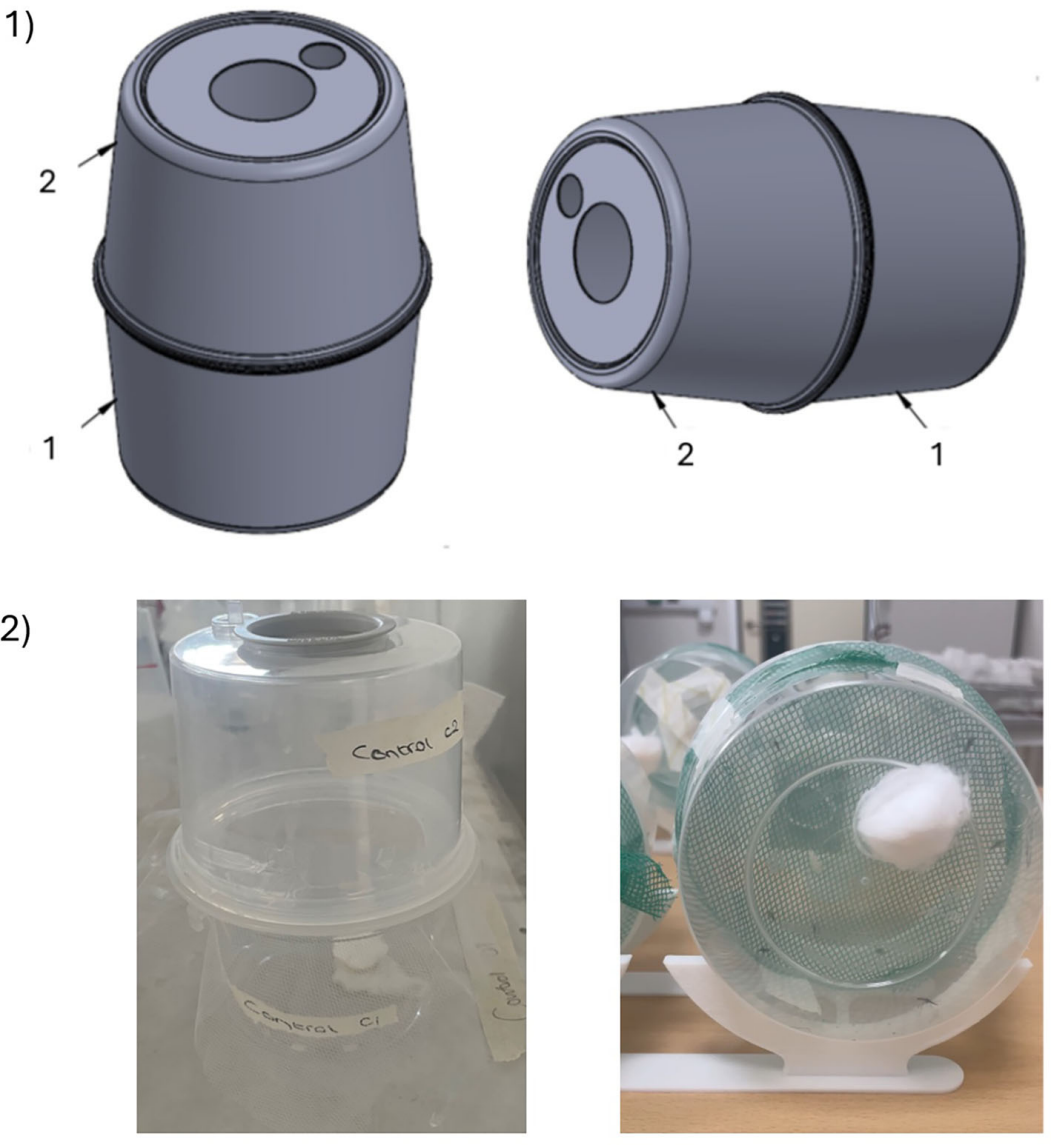
1) Computerised image of the original mini chambers in the vertical position (left) and horizontal position (right). 2) Photographs of the mini chambers in use in the vertical position (left) and horizontal position (right). The net piece is held in position between the two compartments using tape and parafilm.

To reduce the cost of the assay and avoid potential issues with availability, an alternative version was designed using readily available materials. The alternative pot was made up of two plastic pots (AVLASH, Amazon) of 12 × 9.2 × 7.6 cm (450 ml/16 oz internal volume) stacked on top of each other using tape to secure them in place. Parafilm was wrapped around the outside during testing to prevent escapees, similar to the original equipment ( Figure 2). The equipment dimensions are listed in Table 4.

**Table 4.** Comparison of dimensions for the different pots evaluated in the mini chamber test.

Assay	Number of mosquitoes	Response volume	Response volume per mosquito	Surface area of net available
Mini chamber	10	1,178.44 cm ^3^	117.84 cm ^3^	223.4 cm ^3^
Alternative pot	10	934.32 cm ^3^	93.43 cm ^3^	223.4 cm ^3^

### Testing conditions

All bioassays were performed in environmentally controlled testing rooms at LSTM (27 ± 2°C and 70 ± 10% RH). Mosquitoes were aspirated from the rearing cages using a HEPA-filtered aspirator and placed in holding containers for acclimatisation in the testing room for one hour before beginning the test.

### Mini chamber test method

Chambers were either set vertically, with compartment one on top, or horizontally ( Figure 2). Mosquitoes were introduced into compartment one, and in vertical tests, chambers were flipped so that compartment one was at the bottom for the exposure period. Cotton wool soaked with a 10% sucrose solution was added on top of compartment two, held in place with tape or parafilm when the chambers were set up horizontally. At the end of the exposure period, mosquitoes were aspirated using HEPA-filtered mouth aspirators, into cups (one cup per chamber). The tip of the aspirator was changed to avoid cross-contamination between the different net types. A 10% sucrose solution was added on top of the cups and replaced every day for 72-hours while mortality was measured. Mosquitoes that were still alive after 72-hours were put in a freezer until death.

### Establishing key testing parameters

The purpose of these experiments was preliminary development of the method. ^16^ Investigating how changing key testing parameters impacted 72-hour mortality and variability in data between replicates established the robustness of the method and helped finalise the draft SOP ready for validation.

*Number of mosquitoes per chamber*

Endpoints in laboratory bioassays can vary depending on the number of mosquitoes exposed to the test item. ^20^^,^^21^ Here, the impact of mosquito number on outcomes in the mini chamber was evaluated. Following the method described above in a vertical orientation with overnight exposure (16 hours), four chambers of each net type (untreated, pyrethroid-only, and pyrethroid-chlorfenapyr) were set up. An overnight exposure was used to match that of the tunnel test so that we were only changing one parameter at a time. Mosquitoes were aspirated into the chambers at 16.30 and removed the following day at 9.00. To determine the effect of mosquito number, an experiment comprising 12 chambers was replicated twice with Kisumu, in which six chambers contained 10 mosquitoes per chamber and six chambers contained 30 mosquitoes. The experiment was repeated with Tiassalé 13 using 10 and 30 mosquitoes. Based on the similarity of the results between all chambers, the experiment was repeated four times to compare 10 and 20 Tiassalé 13.

*Length of exposure*

A shorter length of exposure would increase throughput and sensitivity on each testing day, while overnight exposure captures the mosquito’s natural nocturnal behaviour. Two different exposure lengths were tested – two hours and four hours – and the results were compared to the data collected from 10 mosquitoes with overnight exposure, as described above. For shorter exposure periods, mosquitoes were aspirated into the chambers at 12.00 midday and removed after two or four hours. These mosquitoes were reared on a shifted light cycle, so all testing was completed during the night phase. On each testing day, 10 mosquitoes were exposed per chamber, with four chambers per net type per day, and for each exposure length (two and four hour), three replicates were completed for Tiassalé 13 and three for Kisumu.

To confirm that the length of exposure was robust to changes in other testing parameters, five replicates were performed using the Tiefora strain in alternative pots. These compared 10 mosquitoes per chamber with 20 per chamber in the new version of the mini chamber. All bioassays had an exposure length of four hours beginning at 12.00 midday. For each replicate, there was one negative control, one pyrethroid-PBO, and four pyrethroid-chlorfenapyr chambers containing 10 or 20 mosquitoes (12 chambers in total).

*Mini chamber orientation*

The tunnel test is performed in a horizontal orientation, but the mini chamber was easier to handle in the vertical position. The ability of mosquitoes to navigate through the net and their resting behaviour on the net may vary with the orientation of the chambers, and the behaviour of mosquitoes around the net will affect their level of exposure and potential mortality, as has been observed in other bioassays. ^21^^,^^22^ On each testing day, 10 ‘light-shifted’ mosquitoes were exposed for four hours beginning at 12.00 midday and ending at 16.00. The 12 chambers were set up as follows: one negative control chamber, one pyrethroid-only chamber, and four pyrethroid-chlorfenapyr chambers in the vertical position, and the same number of chambers in the horizontal position. Four replicates were completed using Kisumu and four with Tiassalé 13.

Further testing was completed to confirm that the previous results were robust to changing the material of the mini chambers and between mosquito strains. These assays were performed using Tiassalé 13 and Tiefora, with overnight exposure in the alternative pot. Ten mosquitoes were exposed overnight in each chamber beginning at 17.00 and ending at 9.00 the following morning. Each day, there was one negative control, one pyrethroid-PBO, and five-seven pyrethroid-chlorfenapyr chambers in the horizontal and the same in vertical positions.

At IHI, two additional replicates were completed, in which one negative control chamber, one pyrethroid-only chamber, and four pyrethroid-chlorfenapyr chambers were in the vertical position, and the same number of chambers were in the horizontal position. Ten pyrethroid-resistant mosquitoes were exposed for four hours beginning at 18.00 and ending at 22.00.

### Comparing the mini chamber test to the WHO tunnel tests

The tunnel test is the current standard practice for testing the bioefficacy of ITNs, where cone tests are not suitable, as is the case for chlorfenapyr-based ITNs. ^11^^,^^14^ To determine the performance of the mini chamber test compared to the current ‘gold standard’, a comparator study was performed. ^16^ The comparator study was conducted at the Ifakara Health Institute (IHI), Bagamoyo, Tanzania, a facility whose team regularly performs WHO tunnel tests to evaluate chlorfenapyr-based ITNs. All comparator bioassays were performed simultaneously and in the same room to minimise variation due to differences in room temperature, humidity, and mosquito rearing.

Tunnel tests were performed as described by Kamande et al. (2022 ^23^). During week one, 10 mosquitoes were exposed in each chamber overnight (16 hours). Six mini chamber pots and six alternative pots were used (one untreated, one pyrethroid-only, and four pyrethroid-chlorfenapyr chambers). Simultaneously, 50 mosquitoes were exposed to ITN samples in the WHO tunnel test overnight (one untreated tunnel, one pyrethroid-only tunnel, and one pyrethroid-chlorfenapyr tunnel). In week two, the same procedure was followed, but with a four-hour exposure period. Each week, three replicates were performed using the pyrethroid-resistant *Anopheles arabiensis* Kingani strain, and one replicate was performed using the pyrethroid-susceptible *Anopheles gambiae* Ifakara strain.

### Validation of SOP with used nets of different insecticidal content

All previous experiments were performed using pieces of new net directly received from the manufacturer. This experiment aimed to test whether the mini chamber was sensitive enough to detect differences in mortality between net samples under different conditions. These nets were from the same batch and had either not been washed (baseline), washed, and used in experimental hut studies (hut used), or nets that had been 20 times washed and used in experimental hut studies (hut washed). These net pieces were provided by IHI after they were generated in a separate study.

On each testing day, one negative control net, two baseline nets, two hut used nets, and two hut washed net pieces were tested with the Tiassalé 13 strain and the same with Kisumu. Ten mosquitoes were exposed overnight in each chamber beginning at 17.00 and ending at 9.00 the following morning. This was repeated three times before the chambers were washed, and three more replicates were completed using the same net pieces and the opposite strain, totalling six replicates per net piece. Concurrently, cone tests were performed on a fifth piece of netting following the WHO standard procedure (WHO, 2024). On each testing day, one set of four cones was used for each treatment group and a negative control with Kisumu and Tiassalé 13. One net piece from each treatment group was used for HPLC analysis.

Based on the results from the overnight exposure, to try and increase sensitivity, a second set of net pieces from the same treatment groups was used to test different exposure lengths, six hours and eight hours. Ten mosquitoes were aspirated into the chambers at 9.00 and aspirated out at 15.00 (6 hour exposure) or 17.00 (8 hour exposure). This was repeated thrice for each exposure length. On each testing day, one set of four cones was used for each treatment group and a negative control with Kisumu and Tiassalé 13. One net piece from each treatment group was analysed using HPLC.

### High performance liquid chromatography (HPLC)

HPLC was performed to determine the concentrations of chlorfenapyr and deltamethrin in the used and unused net pieces of PermaNet Dual. Ten pieces of net were used for the analysis: three unused pieces from the roof section, one unused from the side of the net, three pieces that were used during overnight testing runs, and three pieces that were used during the 4-hour testing. HPLC was also performed on the aged Interceptor ^®^ G2 obtained from IHI for comparison with the mini chamber data to validate its sensitivity to detect a difference in bioefficacy between net samples with different surface and/or total insecticide content. Two ‘baseline’ net pieces, two ‘hut used’ net pieces, and two ‘hut washed’ net pieces were assessed. All net pieces were assessed in triplicates.

Following the methods described by Walker et al. (2022 ^24^), three circles were cut from each net piece and soaked in 100 μg/ml dicyclohexyl phthalate (DCP) in 1:9 1-propanol:heptane for 45 minutes in a dri-block (Techne, Cheshire, UK) at 85 °C. One millilitre of the liquid was evaporated using a dri-block and sample concentrator (Techne, Cheshire, UK) at 60°C under compressed air, resuspended in 1 ml acetonitrile, vortexed at 3000 rpm for one minute and centrifuged at 13,000 rpm for 15 minutes. For quantification, 20 μl of liquid was injected into the HPLC system. The limits of detection (LoD) and quantification (LoQ) for chlorfenapyr were 0.014 and 0.047, respectively. For deltamethrin, the LoD and LoQ were 0.0058 and 0.019, respectively.

### Data analysis

All data were collected on paper record sheets, transcribed into Microsoft Excel, and imported into RStudio (Version 4.5.1 ^25^) for analysis. For each analysis, data were subsetted to include the mosquito strain, and only the data that were relevant to the test were included (for example, when analysing exposure length data, only data where 10 mosquitoes were used was included, the data with 20 and 30 mosquitoes were removed).

To analyse the 72-hour mortality endpoint, a generalised linear mixed model (GLMM) with a binomial link function using the ‘lme4’ package. Net type and the parameter of interest (number of mosquitoes, exposure length, orientation, and method) were included as fixed effects, whereas date was used as a random effect to account for random variation between testing days. As the parameters assessed contained multiple groups (control, pyrethroid-only, pyrethroid-PBO, and pyrethroid-chlorfenapyr), post-hoc analysis was used to assess comparisons between different subgroup combinations. To minimise the risk of false positives during multiple comparisons, Bonferroni corrections were applied to any post-hoc analysis that generated more than three results. The means and confidence intervals presented are the predicted means from the models, generated using a bootstrap method with the ‘bootMer’ function in ‘lme4’.

## Results

### Strain characterisation

The results of the WHO tube bioassays demonstrated that the Tiassalé 13 strain was resistant to pyrethroids (24-hour mortality = 44%) at the WHO diagnostic dose of 0.05% deltamethrin ( Table 5). The Kisumu strain was fully susceptible to 0.05% deltamethrin (24-hour mortality = 100%). In the WHO cone tests, 72-hour mortality was higher in the Kisumu strain than in Tiassalé 13 with PermaNet 2.0 (95% vs. 15%) and PermaNet Dual (63% vs. 10%), as expected.

**Table 5.** Characteristics of pyrethroid resistant and susceptible strains used for the development of the mini chamber test method.

**Pyrethroid resistant mosquito strain: Tiassalé 13**	
Species: *An. gambiae*	
% mortality (24 hours) in WHO tube with deltamethrin (0.05%)	44%
% mortality (72 hours) in WHO cone test with new pyrethroid-only ITN (PermaNet 2.0)	15%
% mortality (72 hours) in WHO cone test with new pyrethroid-chlorfenapyr ITN (PermaNet Dual)	10%
**Pyrethroid susceptible mosquito strain: Kisumu**	
Species: *An. Gambiae*	
% mortality (24 hours) in WHO tube with deltamethrin (0.05%)	100%
% mortality (72 hours) in WHO cone test with new pyrethroid-only ITN (PermaNet 2.0)	95%
% mortality (72 hours) in WHO cone test with new pyrethroid-chlorfenapyr ITN (PermaNet Dual)	63%

### Establishing key testing parameters

*Number of mosquitoes per chamber*

**Figure 3.**
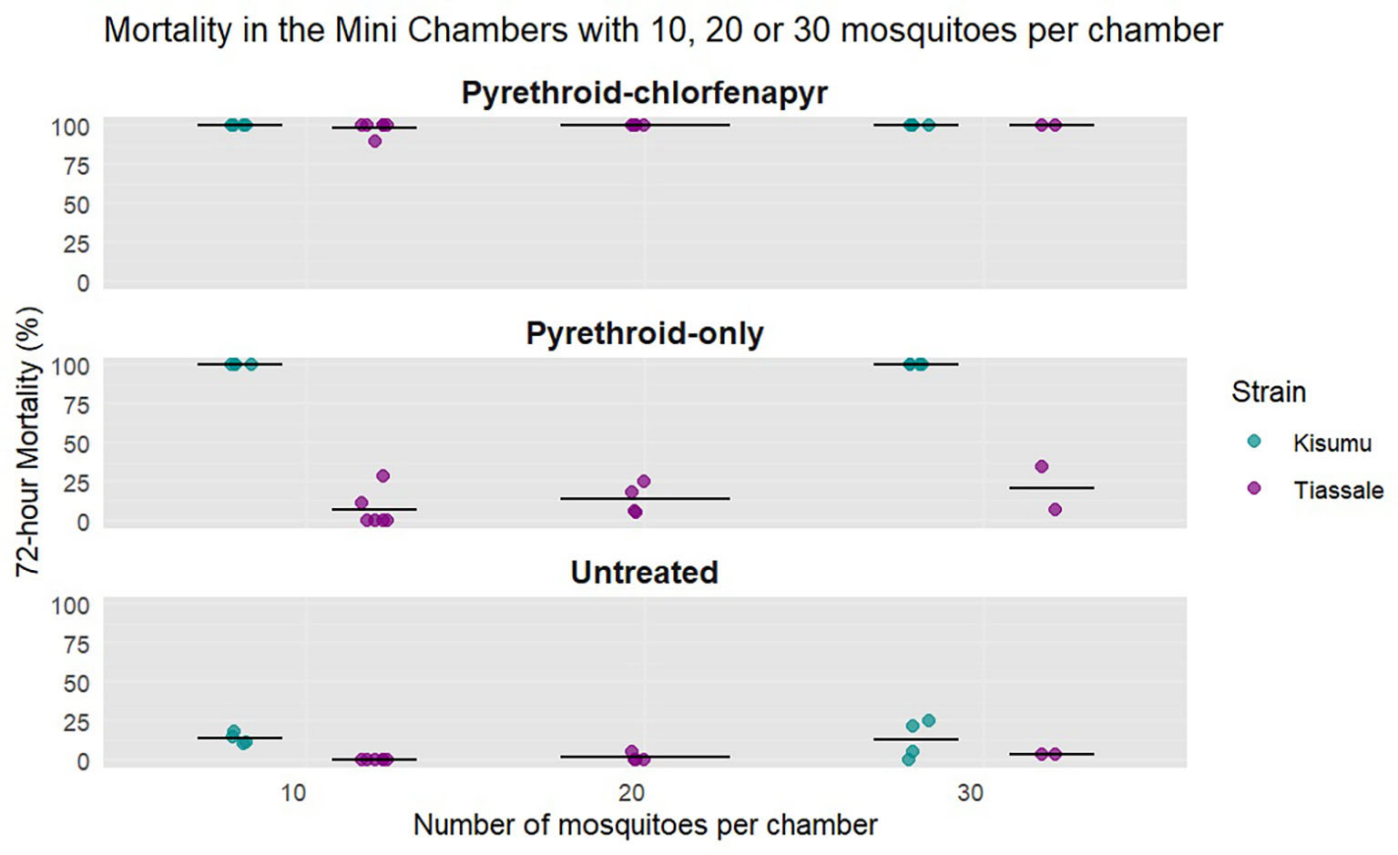
Tiassalé 13 and Kisumu 72-hour mortality rates in the mini chamber test with 10, 20, or 30 mosquitoes per chamber. All mosquitoes were exposed overnight. Individual points are the raw data, and the line indicates the mean percentage 72-hour mortality.

Changing the number of mosquitoes within a single chamber had no significant impact on 72-hour mortality with overnight exposure ( Figure 3); however, it did affect the variability. With 10 Tiassalé 13 mosquitoes per chamber, the mean mortality was 98% compared to 99% with 20 mosquitoes per chamber (p = 1), and 99% with 30 mosquitoes per chamber (p = 1). For the Kisumu strain, mortality was 100% in the pyrethroid-chlorfenapyr and pyrethroid-only chambers with either 10 or 30 mosquitoes (p = 0.99).

Across all net types, the coefficient of variation (CoV) reduced as the number of mosquitoes per chamber increased. The lowest variation was observed in the pyrethroid-chlorfenapyr chambers, ranging from 0 with 20 and 30 mosquitoes to 0.04 with 10 mosquitoes. However, no effect of mosquito density on mortality was observed, and all mosquito densities tested were sufficient to measure the lethal effects of pyrethroid-chlorfenapyr ITNs in the mini chamber. Ten mosquitoes per chamber were selected to maximise the number of net pieces that can be tested with a limited number of mosquitoes.

*Length of exposure*

**Figure 4.**
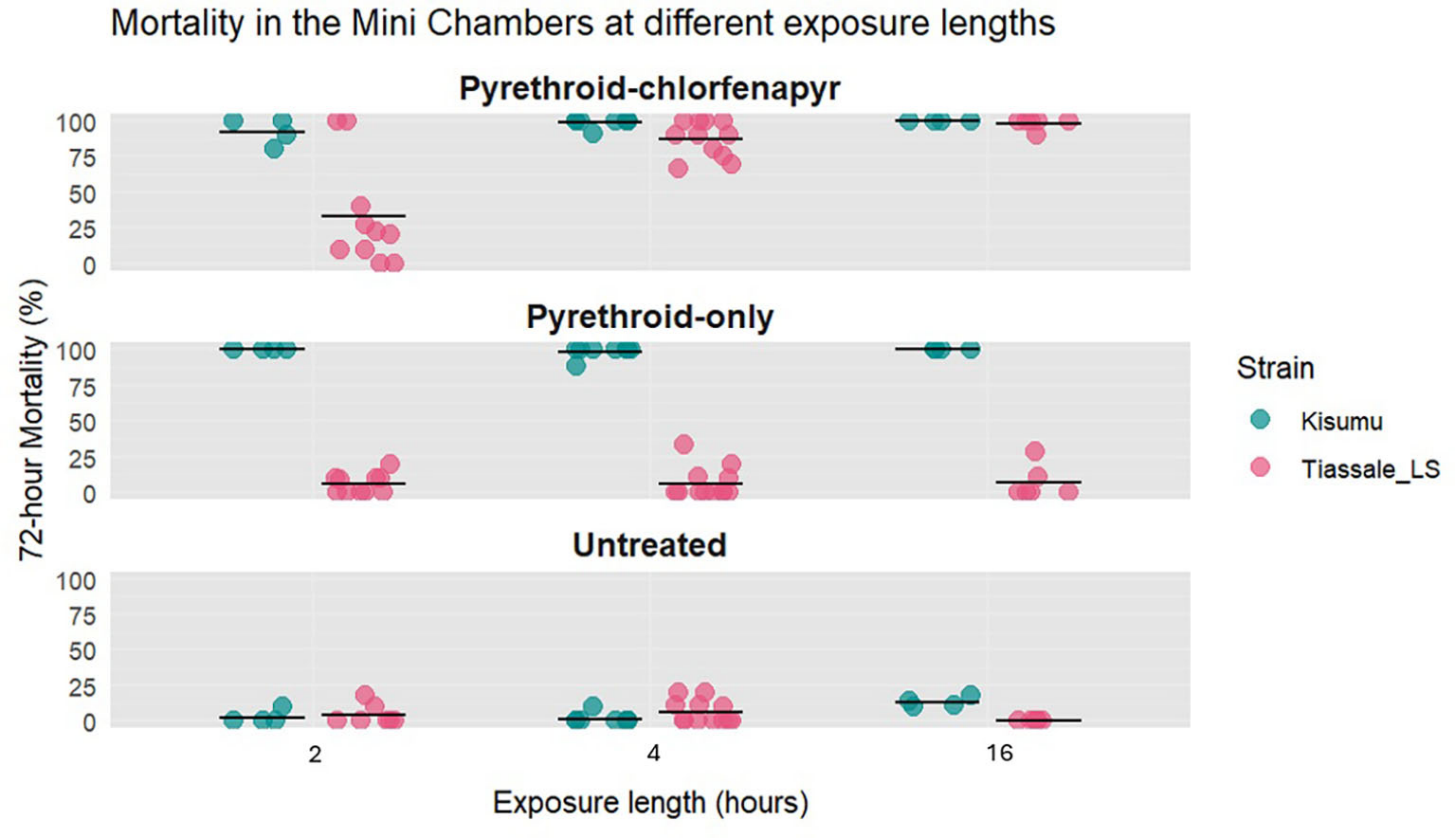
Percentage mortality at 72-hours after two hour, four hour, and overnight exposure in the mini chamber. Tiassale_LS refers to the light-shifted Tiassalé 13 strain. Individual points are the raw data, and the line indicates mean percentage 72-hour mortality.

After overnight exposure, 72-hour mean mortality of Tiassalé 13 mosquitoes in the pyrethroid-chlorfenapyr chambers was 99%. Shortening the exposure reduced the 72-hour mean mortality significantly: two hour exposure resulted in 35% mortality (p = 0.0001) and four hour exposure 74% (p = 0.0054). There was no significant difference in the 72-hour mean mortality between the two hour and four hour exposure lengths (35% vs. 74%, p = 0.008). In contrast, the Kisumu (susceptible) mosquitoes showed consistently high 72-hour mean mortality of 95%-100% across all exposure lengths ( Figure 4).

Decreasing the exposure length increased the coefficient of variability in the pyrethroid-chlorfenapyr chambers. Overnight exposure length (16 hours) had the lowest variability (CoV = 0.04) followed by four hours (0.33) with two-hours having the highest (1.14).

Based on these results, a four-hour exposure was initially selected for the draft SOP to increase testing throughput, as it produced mortality levels broadly comparable to published WHO tunnel tests (53.9%-90%, ^26^^,^^27^) and the data generated during the comparator study.

**Figure 5.**
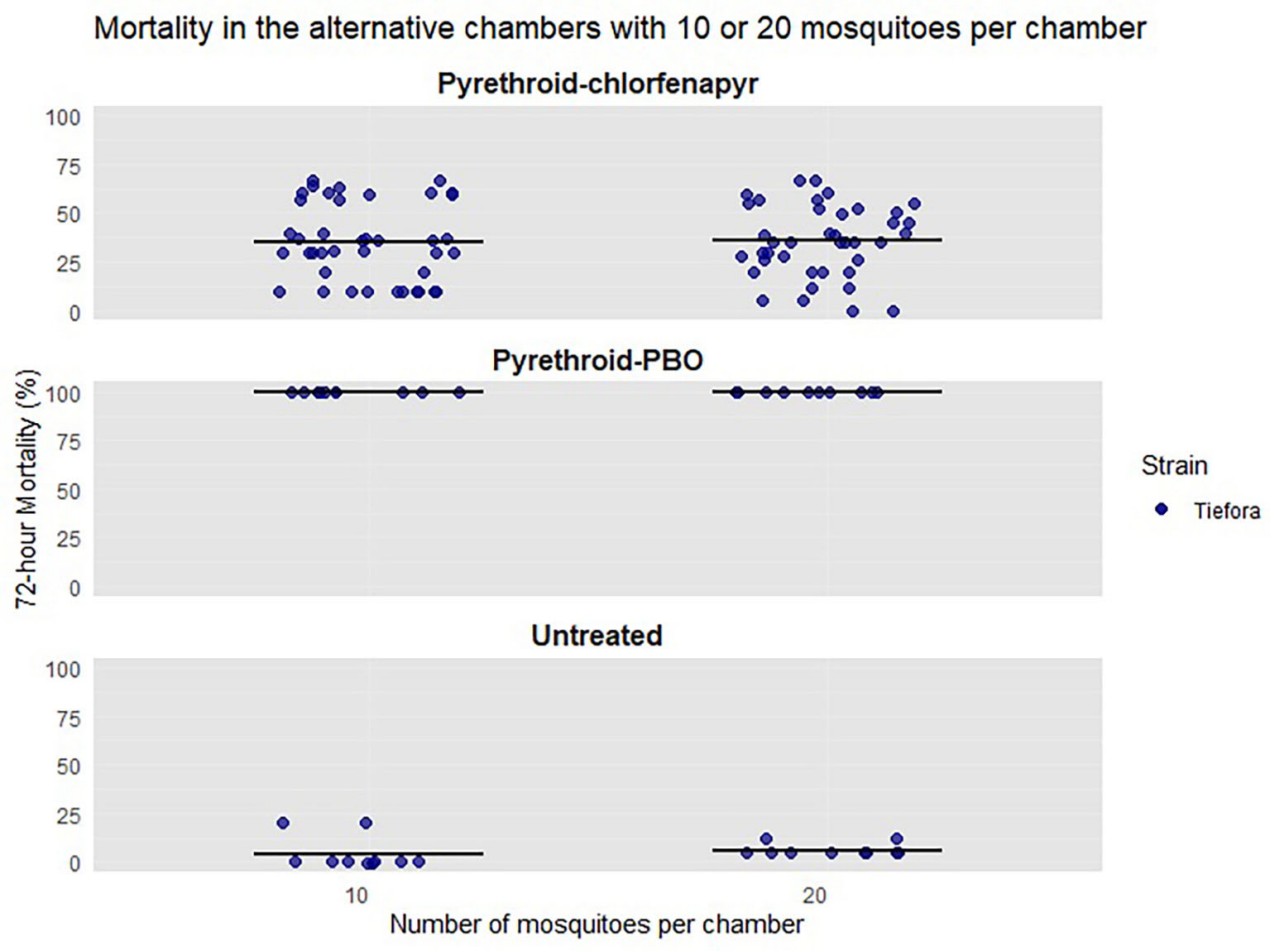
Percentage mortality of light-shifted Tiefora at 72-hours after either 10 or 20 mosquitoes were exposed for four hours. Individual points are the raw data, and the line indicates mean percentage 72-hour mortality.

When the Tiefora strain comparison of 10 and 20 mosquitoes per chamber with a four hour exposure length was completed ( Figure 5), there was no significant difference in the 72 hour mortality (36% vs. 35%, p = 0.84). However, the mortality rate of the Tiefora strain was lower than that of Tiassalé 13 (36% vs. 74%) and the variability was much higher (CoV = 0.52 vs 0.33). This led to a change in the exposure length to overnight.

*Mini chamber orientation*

There was no significant difference in the mean 72-hour mortality when the orientation was changed from vertical to horizontal using Tiassalé 13 (70% vs. 67%, p = 0.60, CoV = 0.28 vs 0.36) or Kisumu (92% vs. 98%, p = 0.02, CoV = 0.05 vs 0.10).

**Figure 6.**
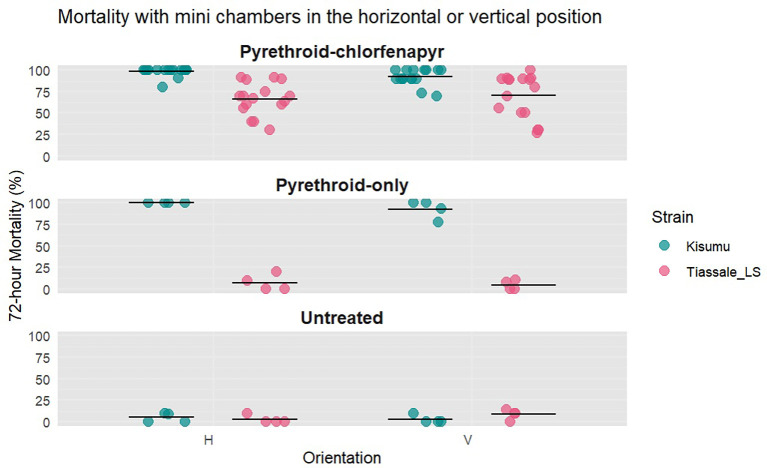
Percentage mortality at 72-hours after 10 mosquitoes were exposed for four hours in either a horizontal (H) or vertical (V) orientation. Tiassale_LS refers to the light-shifted Tiassalé 13 strain. Individual points are the raw data, and the line indicates mean percentage 72-hour mortality.

**Figure 7.**
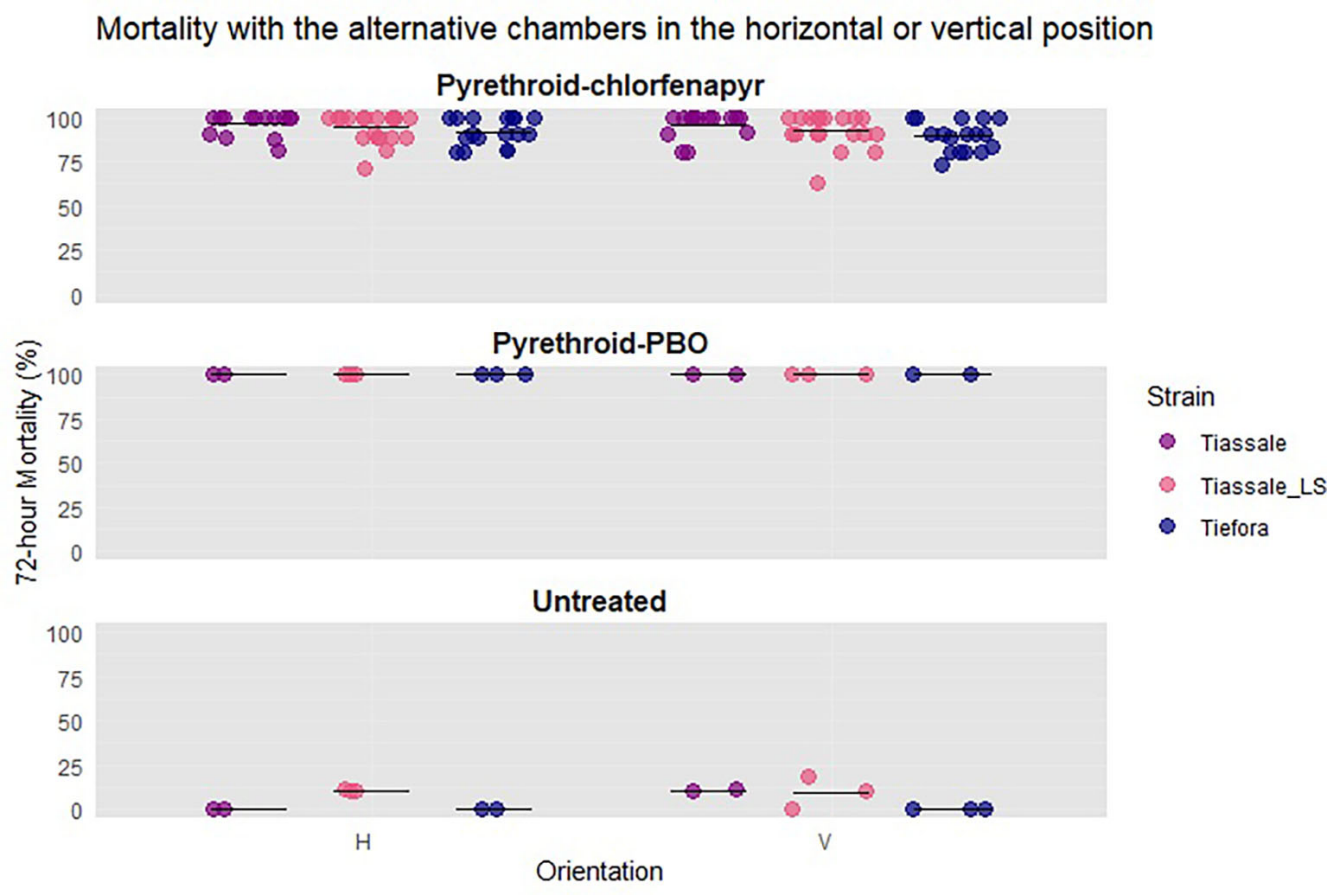
Percentage mortality of standard Tiassalé 13, light-shifted Tiassalé 13 and the light-shifted Tiefora strain. Individual points are the raw data, and the line indicates mean percentage 72-hour mortality.

In overnight comparisons using the alternative pots, there was no difference in 72-hour mortality due to orientation ( Figure 7) with Tiassalé 13 (96% vs. 97%, p = 0.82), ‘light-shifted’ Tiassalé 13 (94% vs. 92%, p = 0.47) or Tiefora (89% vs. 92%, p = 0.17). Variability was lower in all strains compared to the four hour exposures. The horizontal orientation had lower variability across all strains in the pyrethroid-chlorfenapyr chambers (Tiassalé 13: H = 0.06 vs. V = 0.08, ‘light-shifted’ Tiassalé 13: H = 0.07 vs. V = 0.09, Tiefora: H = 0.09 vs. V = 0.1).

**Figure 8.**
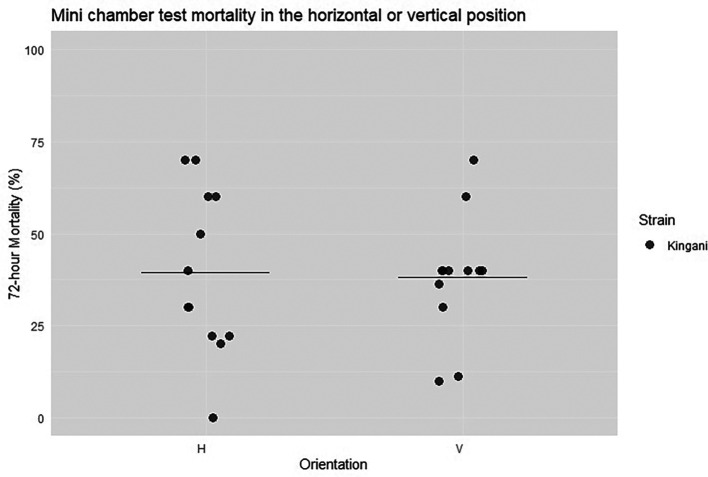
Percentage mortality of Kingani at 72-hours after either 10 mosquitoes were exposed for four hours in a horizontal or vertical orientation. Individual points are the raw data, and the line indicates mean percentage 72-hour mortality.

No significant difference in orientation was observed ( Figure 8) when the alternative chambers were performed at IHI for four hours with the pyrethroid-resistant Kingani strain (40% vs. 38%, p = 0.8, CoV = 0.57 vs 0.44).

Although the vertical orientation has an advantage in terms of ease of testing, this contrasts with the tunnel test orientation and comes with an increased risk of mosquitoes resting on the net piece during the exposure time, rather than simply flying through and being overexposed to the insecticide. For these reasons and because of the lower variability, a horizontal orientation was selected for the draft SOP.

### Comparing the mini chamber test to the current standard practice

**Figure 9.**
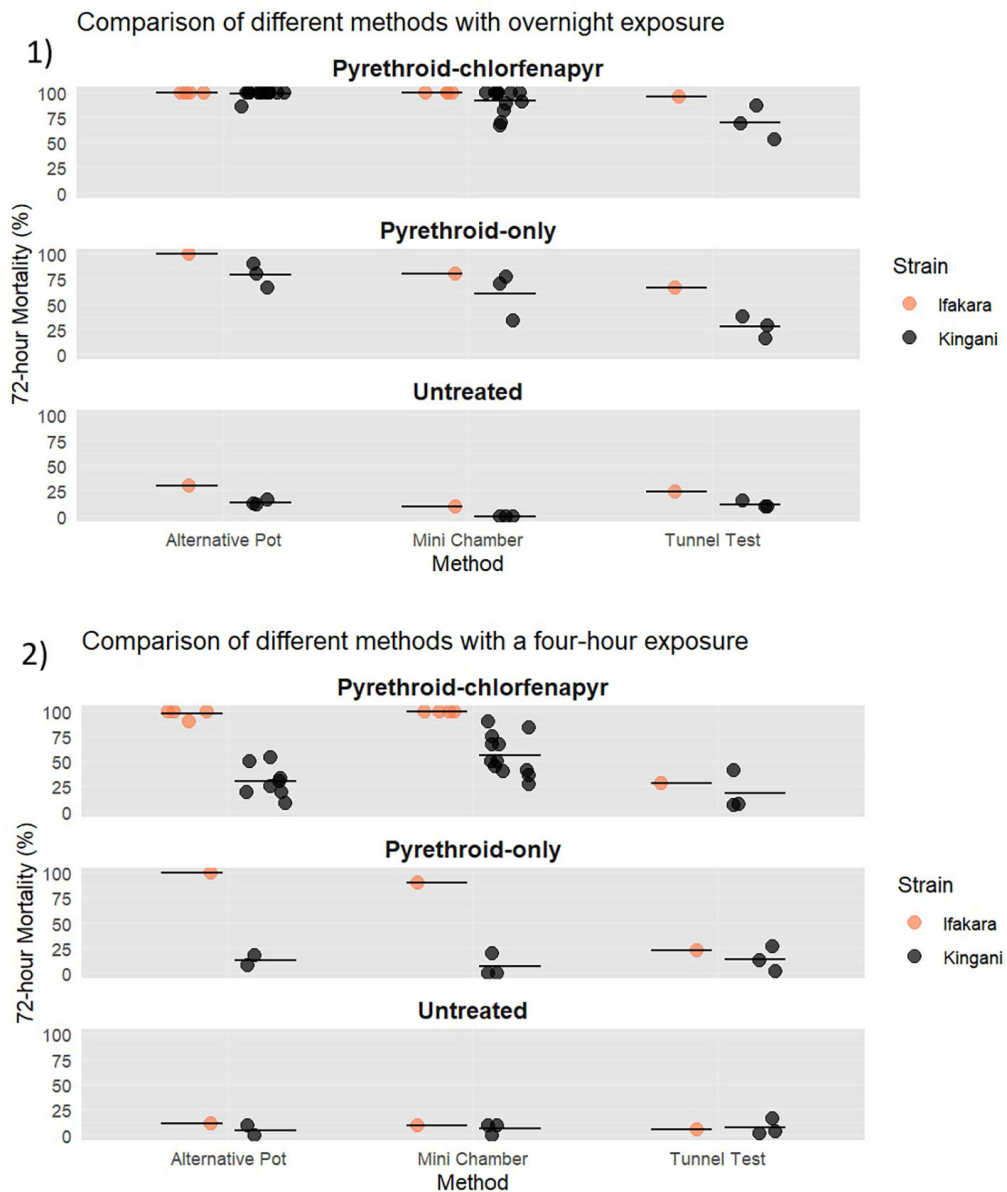
Percentage 72-hour mortality of pyrethroid susceptible and resistant strain in the alternative mini chamber, the mini chamber and the WHO tunnel test. 1) Overnight exposure in all methods. 2) Shortened four hour exposure in all methods.

The original mini chamber equipment, alternative pots, and tunnel tests were compared using both four-hour and 15-hour (overnight) exposures. When the bioassays were performed with overnight exposure, mortality was >90% in the mini chamber and alternative pots and 70% in the tunnel test with the pyrethroid-chlorfenapyr nets ( Figure 9). When exposure was shortened to four-hours, mortality in all bioassays decreased, with mini chamber mortality down to 56%, alternative pot 30%, and tunnel test 15%. The variability was highest in the pyrethroid-chlorfenapyr tunnels at both exposure times compared to the pyrethroid-chlorfenapyr chambers. With a four hour exposure length, the tunnel test had a CoV of 1.05, the original mini chamber was 0.35, and the alternative pot was 0.51. With an overnight exposure length, the tunnel test had a CoV of 0.24, the original mini chamber was 0.14, and the alternative pot was 0.04.

### Validation of SOP with used nets of different insecticidal content

**Figure 10.**
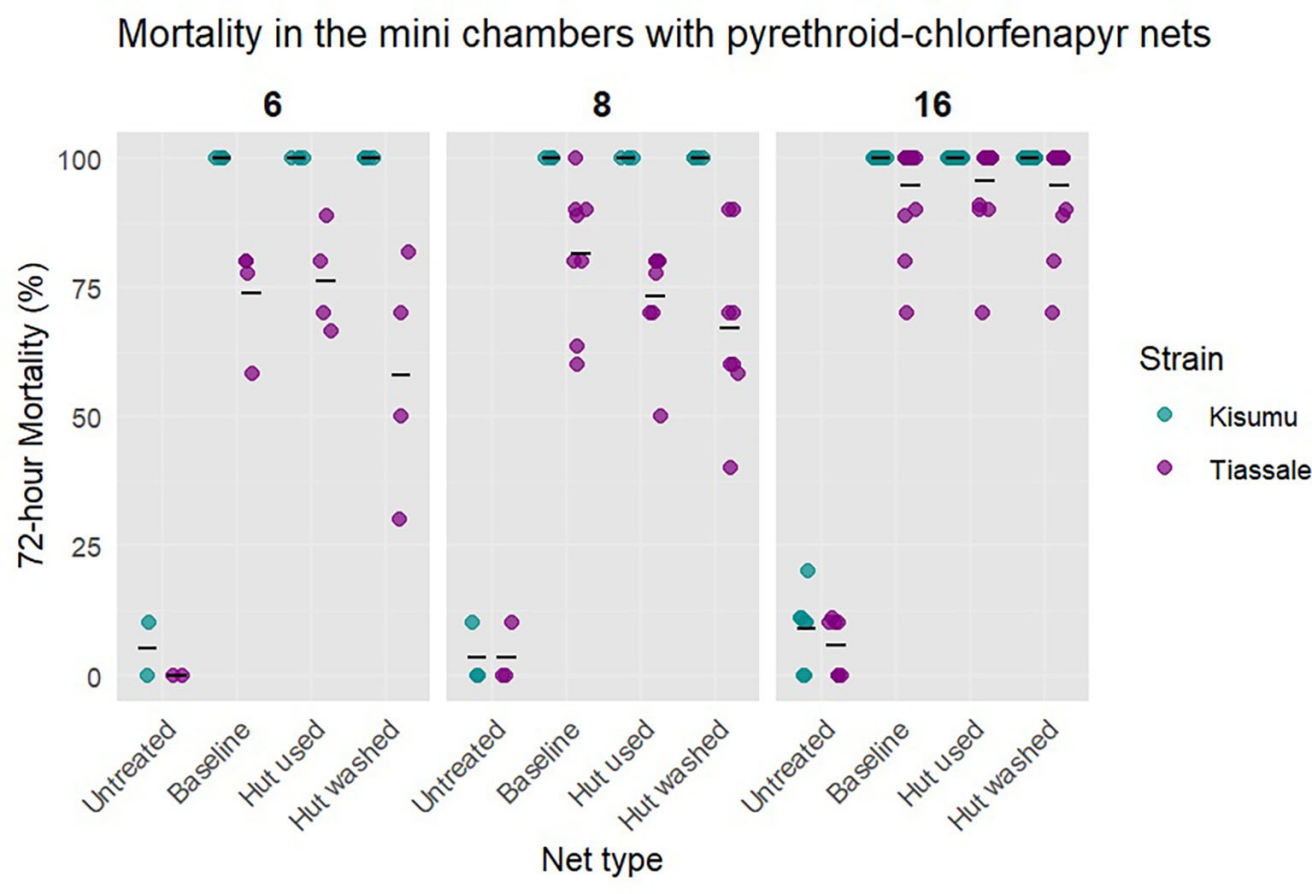
Percentage mortality of Tiassalé 13 and Kisumu when exposed to net pieces from three different conditions. Baseline refers to unwashed and unused net pieces, Hut used refers to unwashed pieces used in an experimental hut study and hut washed refers to the pieces that were washed and used in an experimental hut study.

The mini chamber test SOP that was drafted after the test parameters were set based on testing with new net pieces was found not to be sensitive enough to detect differences in mortality between the baseline, hut used and hut washed nets ( Figure 10). The 72 hour mortality was consistently high in Kisumu (100% across all net types) and Tiassalé 13 exposed to all net samples (Tiassalé 13: 95% (91%-98%), 96% (93%-99%), and 95% (91%-99%), p = 0.9). The variability with an overnight exposure length was 0.1 for the baseline and hut washed nets and 0.09 with the hut used nets. Different exposure lengths were tested to increase the sensitivity.

Tests with six and eight hour exposure lengths were completed using mosquitoes reared with a standard light schedule. We found that shortening the exposure to eight hours produced 81% (95% CI: 71%-91%) mortality with the baseline nets, 73% (95% CI: 62%-84%) with the hut used nets, and 67% (95% CI: 54%-79%) with the hut washed nets. There was no significant difference between the baseline nets and hut washed nets (p = 0.3). Shortening further to six hours produced 72% (95% CI: 56%-85%) mortality with the baseline nets, 75% (95% CI: 60%-89%) with the hut used nets, and 57% (95% CI: 38%-74%) with the hut washed nets ( Figure 10). There was no significant difference in mortality between the baseline and hut washed net groups (p = 0.1). At both exposure lengths, the hut washed nets had the highest CoV (six hours = 0.39, eight hours = 0.25). The CoV for the baseline nets at six hours was 0.14 and at eight hours 0.13. For the hut used nets, the eight hour CoV was 0.17 for the baseline nets and 0.14 for the hut used nets, respectively.

When the bioassays were performed, the conditions and insecticidal contents of the net pieces were unknown. HPLC was performed to determine the insecticidal content of the nets and to aid in determining the most sensitive exposure length. Based on the HPLC results ( Figure 12), we would not expect to see a difference in mortality between the baseline net pieces and the hut used net pieces. To increase the sensitivity of the bioassay while keeping the method logistically accessible to as many laboratories as possible, a six hour exposure length was selected for the draft SOP for internal validation.

### High-performance liquid chromatography

**Figure 11.**
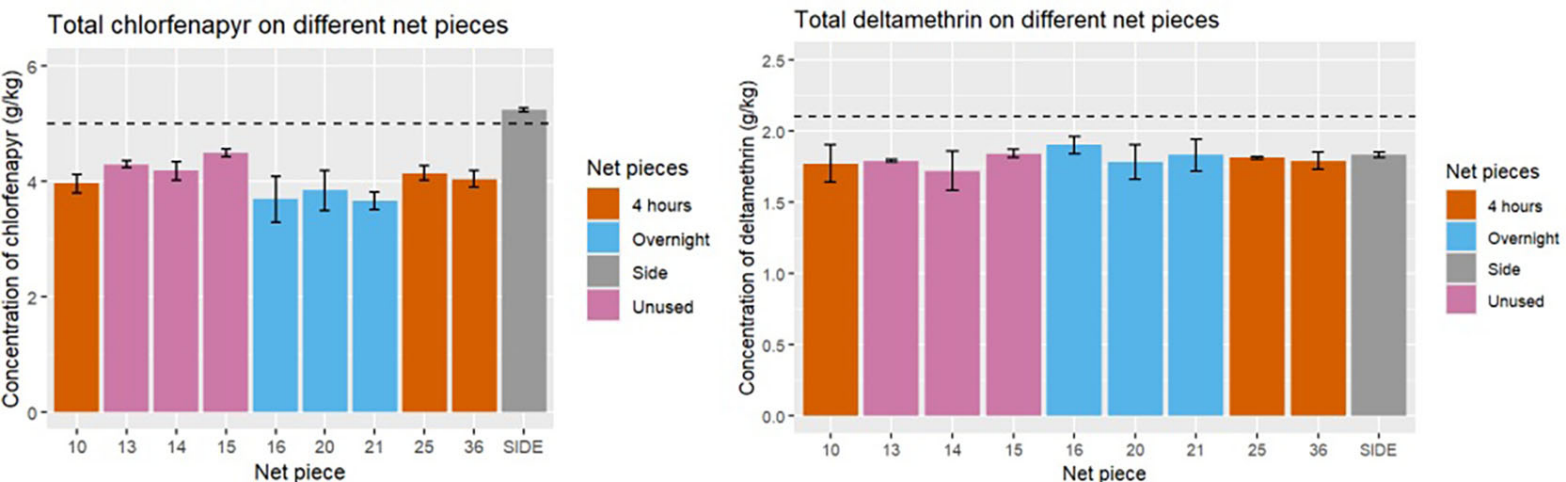
Left) The total concentration of chlorfenapyr on each net piece from HPLC analysis. The dotted line represents the loading dose of chlorfenapyr on PermaNet Dual nets, 5 g/kg. Right) The total concentration of deltamethrin on each net piece from HPLC analysis. The dotted line is the loading dose of deltamethrin in PermaNet Dual nets, 2.1 g/kg.

HPLC analysis was performed to determine the total deltamethrin and chlorfenapyr content on a total of 10 net pieces from the same net as was sampled for mini chamber testing, for comparison with the bioassay results and validation of the mini chamber as being able to discriminate between net samples with different total and/or surface insecticide content. The mean content of chlorfenapyr on net pieces which had not been used for mini chamber testing was 4.3 g/kg, on net pieces used for overnight exposures was 3.7 g/kg, on net pieces used for four hour exposures 4.0 g/kg ( Figure 11) and unused side pieces was 5.2 g/kg. The mean content of deltamethrin in all net pieces was 1.8 g/kg. All net pieces, regardless of use, were within the 25% allowance for insecticidal content on an ITN.

**Figure 12.**
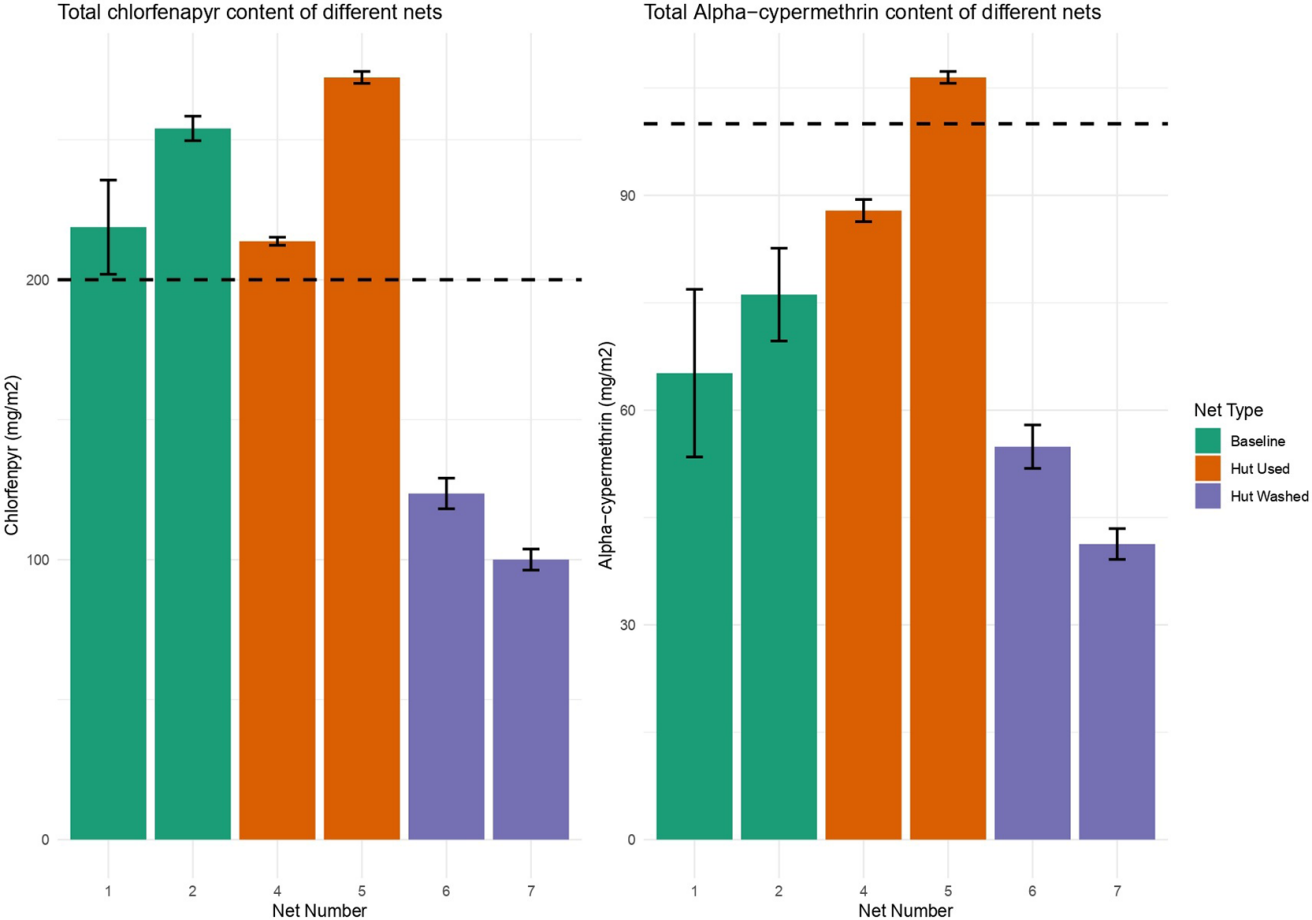
Left) The total concentration of chlorfenapyr on each net piece from HPLC analysis. The dotted line represents the loading dose of chlorfenapyr, 200 mg/m ^2^. Right) The total concentration of alpha-cypermethrin on each net piece from HPLC analysis. The dotted line is the loading dose of alpha-cypermethrin, 100 mg/m ^2^.

HPLC analysis was also performed on the net samples provided by IHI to measure the amount of remaining insecticide in each treatment group, measuring both the total insecticide content and quantifying the insecticide on the surface of the nets by analysing a solvent wash. The mean content of chlorfenapyr on baseline net pieces was 236 mg/m ^2^, on hut used net pieces was 242 mg/m ^2^ and on hut washed net pieces was 111 mg/m ^2^ ( Figure 12). The mean content of alpha-cypermethrin in baseline net pieces was 71 mg/m ^2^, in hut used net pieces was 97 mg/m ^2^ and in hut washed pieces was 48 mg/m ^2^.

### Final SOP

The mini chamber draft SOP, ready for validation, is provided in the Github repository ( https://github.com/i2i-Data-Repository/Mini-Chamber-Test-paper-data-R-code-and-SOP). The established testing parameters were 10 mosquitoes per chamber in the horizontal position with a six hour exposure length. The variability associated with this SOP ranges from 0.14-0.39 with the Tiassalé 13 strain, when a single batch of nets is tested. Based on further validation of the method, the SOP may be adapted, and this process can be guided by the generation of data through application of the method to different batches of net samples of different conditions and in different use cases.

## Discussion

Pyrethroid resistance in malaria vectors threatens the effectiveness of malaria control in sub-Saharan Africa. To combat this problem, new insecticides such as chlorfenapyr have been introduced into dual AI ITNs. Given that WHO cone tests cannot be used for laboratory-based bioefficacy testing of chlorfenapyr ITNs, ^11^ the WHO tunnel tests are currently the best available test method. These bioassays are not accessible to all researchers because they require large numbers of mosquitoes (50 per tunnel ^23^) and an animal host, which creates a problem for assessing chlorfenapyr ITNs. To improve the rate of net testing and accessibility to bioassays, the mini chamber assay was developed to measure mortality in susceptible and resistant strains of *Anopheles* exposed to chlorfenapyr-treated ITNs.

Experiments were performed to optimise this bioassay by varying the number of mosquitoes per chamber, exposure length to the ITN, and orientation of the chambers and comparing results, which have previously been identified as important influencers of mortality scores in other bioassays. ^16^^,^^21^^,^^28^ These parameters were tested with a range of pyrethroid-susceptible and resistant *Anopheles* strains to determine the robustness of the mini chamber test.

The number of mosquitoes in a bioassay is often correlated with mortality, because with a higher density of mosquitoes in the testing arena, there may be more activity due to a crowding effect that disturbs the resting behaviour of the mosquitoes. For all insecticides, this is likely to increase exposure, but for chlorfenapyr, this is especially relevant, as more movement leads to greater metabolic activity and higher rates of conversion of the pro-insecticide to active forms. In our experiments, however, no significant effect of mosquito number was observed, with 10-30 mosquitoes per mini chamber tested, as seen in the tunnel test. ^23^

The mini chamber had a response volume of 1,178.44 cm ^3^ while the alternative pot had a response volume of 934 cm ^3^. The tunnel test had a response volume of 37,500 cm ^3^ giving each mosquito a response volume of 750 cm ^3^ while the original pot and alternative had a response volume of 117.84 cm ^3^ and 93.43 cm ^3^ per mosquito, respectively. The surface area of the net available for mosquitoes to contact in the mini chambers was 223.4 cm ^2^ and in the tunnel test was 617.9 cm ^2^.

Varying the length of exposure to the net piece greatly impacted 72-hour mortality. Overnight exposures had consistently high 72-hour mortality, which was expected with brand-new nets from the manufacturer. However, when testing nets that had been used and stored at 4°C, the overnight exposure length was not sufficiently sensitive to detect differences between the nets. This was a result of the higher mortality with longer exposure, which may be an artefact in that mosquitoes do not just encounter the net during normal behaviour but are subject to prolonged and effectively forced contact.

The selection of the best exposure length is a balance between practical and biological considerations. An exposure length of 4 hours would allow the bioassays to be run easily on a single testing day, benefitting throughput and convenience relative to longer exposure lengths and overnight tunnel testing. However, while a 4 hour exposure produced a similar mean 72 hour mortality to results previously reported for chlorfenapyr-treated nets in the WHO tunnel test, ^27^^,^^29^ there was high variability in data generated with a 4 hour exposure, and the same level of mortality was not observed when the mini chambers were performed during the day at IHI.

Chlorfenapyr is most active when mosquitoes are metabolically active in the evening. ^30^^,^^31^ Light-shifting rearing and testing is possible at some facilities, however not all. Replicate testing was performed at IHI with exposure between 18.00 and 22.00; however, this did not increase 72 hour mortality substantially. Exposure lengths of 6 and 8 hours were assessed with used nets in an attempt to improve the sensitivity of the mini chamber test relative to overnight exposure while keeping it as a bioassay that can be performed during standard working hours. We used mosquitoes reared on a standard light cycle and found that a 6 hour exposure length delivered mortality data that showed the same trend as the HPLC results, suggesting that further optimisation of the mini chamber method could provide a proxy measure for insecticide content on a net. A 6 hour exposure is logistically easier within a normal working day. Although 6 hours was selected based on the results of testing aged nets, it is likely to be a suitable exposure length for new nets, since we did not observe a difference in 72h mortality between 4 hour and 16 hour exposures with pyrethroid-chlorfenapyr nets.

Originally, the mini chambers were adapted from BugDorm Insect Breeder Pots (MegaView Science, Taiwan) however to improve the availability of the mini chamber test an alternative version was tested to determine whether similar levels of mortality were seen in commercially bought deli pots that are more widely available. When the original mini chamber, alternative pot, and tunnel test were used simultaneously, there were significant differences in 72 hour mortality between the mini chamber and the alternative pot. To combat these differences, all parameters were retested in the alternative pot to ensure the robustness of the mini chamber test. Both versions have an advantage over the tunnel test in terms of the laboratory bench space required per replicate.

After optimising the mini chamber method using new nets and a range of mosquito populations, it was important to use the method to test nets with different insecticide contents to demonstrate the sensitivity of the bioassay method to detect these differences. Mortality in the susceptible Kisumu strain was 100% for all nets regardless of exposure length, which is not typically achieved in cone tests with chlorfenapyr-treated nets ( Table 5), demonstrating that the method is an improvement on the available laboratory methods, but also that it is essential to use pyrethroid-resistant mosquitoes to measure the efficacy of dual-active nets with any sensitivity. ^19^ Mortality in the resistant Tiassalé 13 strain was very similar with all net samples regardless of whether they had been used or washed with an overnight exposure, but the sensitivity of the assay increased with a shorter exposure length, and the mortality results best matched the HPLC measures of remaining insecticide content on the nets with a 6 hour exposure length. Although the trend of 72h mortality between treatments matched the HPLC results, we were unable to detect a significant difference in mortality between treatments.

Further work is therefore needed to optimise the mini chamber method to improve sensitivity through testing with additional batches of nets. The used and washed net samples for this study were obtained opportunistically; therefore, there are some questions about the effect of long-term storage or shipping conditions on the chemical content and bioefficacy. Further studies with net samples prepared for this purpose in a more controlled manner would be helpful to further demonstrate the ability of the mini chamber to act as a sensitive proxy for the analysis of chemical content.

There are several steps to the validation of bioassay methods, ^16^ and this study represents the preliminary development and feasibility experiments, exploring the robustness and reliability of the method, and demonstrating that the method is scientifically sound, repeatable, and efficient. We have shown its suitability for testing bioefficacy in new nets, testing for differences between bioavailable AI content on aged nets, and testing with different strains of pyrethroid-resistant *Anopheles* mosquitoes. For full validation, an internal validation, based on the data and SOP generated by this study, would define the performance characteristics and compile a data package to be used in external validation. Separate validation studies for different use cases might be needed, for example, residual efficacy testing of used nets versus batch testing of new nets. An external validation would then define the performance at a minimum of two external laboratories and measure between laboratory variation. As part of the formal validation of the mini chamber method, it would benefit from further testing with nets of different conditions and from different batches to optimise and improve sensitivity in detecting small differences in chemical content.

In the meantime, we hope that laboratories can use the SOP provided here to measure the bioefficacy of new and washed pyrethroid-chlorfenapyr nets. Method validation would be supported by the application of the method in real-world settings and by reviewing the data generated against conventional analysis.

## Conclusion

The aim of this study was to demonstrate the potential of the mini chamber test, a novel bioassay for evaluating the bioefficacy of chlorfenapyr-treated ITNs. This method has been shown to be an alternative to the cone test in measuring mortality in exposed mosquitoes to measure bioefficacy, which is not suitable for testing a slow-acting pro-insecticide. The results were comparable to those of the tunnel test, thus freeing bioefficacy testing from the restrictions imposed by the requirement of an animal host. The parameters were set using new net samples. While the results of testing the method against a set of nets aged in different conditions were promising, further optimisation is needed to ensure that the method is sensitive enough to distinguish between samples with differing residual insecticide contents. For the mini chamber test to be used with confidence and to meet the requirements of the WHO Prequalification of Vector Control Products team, the method must be formally validated.

Presented here are the results of the preliminary development stage of the Method Validation Framework of Matope et al. (2023 ^16^) and a standard operating procedure (SOP) has been drafted ready for feasibility studies to be completed and internal and external validation to be conducted. Once validated, this method is expected to provide a viable alternative to cone and tunnel tests for evaluating the efficacy of chlorfenapyr ITNs. The mini chamber could be applied during product development, durability monitoring, decision making about targeting ITN deployment, and to generate data to calculate the wash retention index (WRI) and regeneration time of a chlorfenapyr-based ITN.

## Data Availability

This project contains the following underlying data: https://doi.org/10.5281/zenodo.17780455. ^32^ The data are available under the terms of the Creative Commons Attribution 4.0 International license (CC-BY 4.0).

## Software availability

Source code available from: https://github.com/i2i-Data-Repository/Mini-Chamber-Test-paper-data-R-code-and-SOP

The source code is available under GNU General Public License version 3 (GPL-3.0-only) ( https://opensource.org/license/gpl-3-0).
